# Genetic analysis of *DDR1* polymorphisms as robust markers for milk yield in Simmental cattle

**DOI:** 10.5194/aab-68-663-2025

**Published:** 2025-11-04

**Authors:** Gülüzar Şengül, Memiş Özdemir

**Affiliations:** 1 Department of Zootechnics and Animal Nutrition, Faculty of Veterinary Medicine, Bingöl University, 12000 Bingöl, Türkiye; 2 Department of Biometrics and Genetics, Faculty of Agriculture, Atatürk University, 24240 Erzurum, Türkiye

## Abstract

This study aimed to determine new polymorphisms in 15 exon regions of the discoidin domain receptor tyrosine kinase 1 (*DDR1*) gene. A total of 72 Simmental cattle were used in this study. Single nucleotide polymorphisms (SNPs) of gene regions were performed using polymerase chain reaction (PCR) high-resolution melting (HRM) analysis followed by sequence analysis. The identified *DDR1* gene regions include the g.28183947 G/T polymorphic region for the 1^′^ exon region of the *DDR1* gene, g.28183480 G/C and g.28183434 C/A-G polymorphic regions for the 3^′^ exon region of the *DDR1* gene, g.28181525 C/A polymorphic region for the 5^′^ exon region of the *DDR1* gene, g.28180846 G/C polymorphic region for the 6^′^ exon region of the *DDR1* gene, g.28180437 C/G polymorphic region for the 7^′^ exon region of the *DDR1* gene, g.28178065 G/C polymorphic region for the 10^′^ exon region of the *DDR1* gene, g.28175989 G/T polymorphic region for the 12^′^ exon region of the *DDR1* gene and g.28173956 T/G and g.28173954 T/C polymorphic regions for the 16^′^ exon region of the *DDR1* gene. As a result of the statistical analysis, a significant relationship was found between the g.28183947 G/T polymorphic region in the 1^′^ exon region of the *DDR1* gene and the 305 d milk yield ( P<0.05 ). A significant relationship was found between the g.28181525 C/A polymorphic region in the 5^′^ exon region of the *DDR1* gene and daily average milk yields, lactation milk yields and the 305 d milk yield ( P<0.05 ). No significant relationship was found between the other polymorphic regions detected and milk yields ( P>0.05 ). Consequently, the genotype and allele gene frequencies of the detected new polymorphic regions were determined. This study suggests that *DDR1* gene polymorphic regions found to affect milk yield in Simmental cattle can be used as new candidate molecular markers in animal breeding and selection.

## 1 Introduction

In recent years, advances in molecular genetics have enabled the identification of a large number of genetic markers associated with genomic regions influencing economic traits in livestock (Dekkers, 2004). The low *heritability* of economically important traits such as milk yield, their polygenic inheritance and the fact that the genotype does not adequately reflect the phenotype have limited the success of selection methods (Gürses and Bayraktar, 2014). In addition, the fact that these traits are often sex limited, are influenced by environmental factors and some traits only being measured in adult animals increase the generation interval and reduce the annual genetic progress (Lara et al., 2002). The use of molecular markers to improve quantitative traits can eliminate the disadvantages of classical selection methods (Özdemir and Doğru, 2008). Through this application, the selection process can be accelerated, the quality of yields can be increased and production costs can be reduced (Fındık and Özdemir, 2022).

Genome-wide association studies (GWAS) have made it possible to screen for SNPs in genes associated with milk production traits in dairy cattle (Ma et al., 2021). SNPs in candidate genes are suitable markers to be used for the identification of causal loci either directly or indirectly associated with quantitative traits (Raschia et al., 2018). DNA markers have been used for the identification of quantitative trait loci (QTL) required for genomic selection, the diagnosis of genetic diseases in animals and parental identification. The implementation of these molecular techniques has provided significant advantages in situations where conventional breeding methods are insufficient, enabling the effective resolution of complex challenges, particularly the accurate and reliable verification of pedigree information (Hillel et al., 1992; Van Arendonk et al., 1994; Nicholas, 2009).

Analyses of animal databases show that approximately 345 QTLs are associated with milk traits, and 71 are associated with mastitis-related traits. As of April 2024, there were 191 181 cattle QTLs in the cattle QTLdb representing 642 key traits, 347 trait variants and 345 cattle genes compiled from 1165 publications. Of these, it was reported that there were 7411 QTLs associated with milk yield, 11 785 QTLs associated with milk fat percentage, 9088 QTLs associated with milk fat yield, 9663 QTLs associated with milk protein percentage and 3676 QTLs associated with milk protein yield (https://www.animalgenome.org/cgi-bin/QTLdb/BT/summary, last access: 28 April 2024).

*DDR1* is a discoidin domain receptor (DDR) protein (Johnson et al., 1993; Perez et al., 1994), which is the extracellular domain subunit of receptor tyrosine kinases that regulate the growth, differentiation and metabolism of cells and play a role in communication with the cellular microenvironment. The *DDR1* gene contains 18 exons on chromosome 23 in cattle (https://www.ncbi.nlm.nih.gov/gene/534092, last access: 20 April 2024). In multicellular cells, the extracellular matrix serves as scaffolding for cells and tissue compartments. To enable communication between tissues and cells, a large number of cell surface receptors are activated by components of the extracellular matrix and soluble ligands. A large family of these surface receptors transduce signals using a tyrosine kinase function. The DDR subgroup differs from other members of the receptor tyrosine kinase family due to the presence of discoidin homologous repeats within various other transmembrane and secreted proteins in their extracellular domains (Vogel, 1999). *DDR1* is a non-integrin collagen receptor, a member of the receptor tyrosine kinase family. Collagens are one of the most abundant proteins found in animals. *DDR1*, one of the three types of collagen receptors, is expressed in the mammary glands, epithelial cells, colon mucosa, brain, lungs and kidneys (Alves et al., 1995). In rapidly growing invasive tumours of the breasts, lungs and ovaries, elevated levels of *DDR1* and *DDR2* are observed, which are consistent with increased proliferative rates and MMP production (Dai et al., 2023).

Milk yield miRNA regulatory networks are controlled by the central genes *DDR1* and *DDHX1* (Yoshitha et al., 2025). *DDR1*, activated by binding to different collagen types, serves not only as a crucial collagen receptor for intracellular signalling related to cell proliferation, survival and colonisation but also plays an important role in the development, growth and regulation of organs (Elango et al., 2022). In order to investigate the effect of the *DDR1* gene on the proliferation, apoptosis and cell cycle of mammary epithelial cells in dairy cows, it was observed that intervention with *DDR1* significantly inhibited cell proliferation, while the proportion of early and late apoptotic cells was significantly increased. Conversely, the overexpression of *DDR1* was found to significantly enhance cell proliferation and substantially inhibit late apoptosis of the cells (Hua et al., 2020).

Facilitated DDR involves cellular functions such as cell survival, cell migration, proliferation and differentiation as well as the remodelling of extracellular matrices (Leitinger, 2014). The deletion of *DDR1* in mice causes significant changes in tissue structure and lactation arrest. This suggests that *DDR1* has an important role in mammary gland function (Vogel, 2001). *DDR1*, which is also a component of human milk, participates in cell-to-cell interaction mechanisms related to cell development and plays a role in their identification (D'Alessandro et al., 2010). *DDR1* is an important mediator of stromal–epithelial interactions during mammary duct formation. Therefore, *DDR1* is essential for female mammals to develop functional mammary glands (Vogel et al., 2001; Juskaite et al., 2017). In experiments with tissue culture, Faraci-Orf et al. (2006) reported that mammary gland epithelial cells require collagen and other basement membrane proteins to carry out prolactin-induced milk protein transcription. In addition, they reported that the *DDR1* pathway synergises with the PrlR pathway in maintaining the STAT5 activation level during mammary gland function (2006). GWAS conducted in Canadian Holstein cattle identified SNPs in the *DDR1* gene located on chromosome 23, and it has been defined as a potential candidate gene for resistance to enzootic bovine leukosis (Bongers, 2023).

The present study aimed to detect molecular marker polymorphisms within selected exon regions of the *DDR1* gene in Simmental cattle and to evaluate the associations between these polymorphic sites and key production traits, including 305 d milk yield, total lactation yield and average daily milk yield. The primary objective of this study is to elucidate the significance of the relationship between the polymorphic regions of the *DDR1* gene and milk yield at the molecular level and to provide scientific contributions to animal breeding and selection programmes based on the findings.

Most of the genetic traits of economic importance in cattle result from quantitative variation. Identifying and validating quantitative traits such as milk yield is costly; however, due to the time-saving and cost-effective nature of such approaches, numerous studies have recently been conducted at the molecular level. The use of molecular markers in cattle breeding is highly significant for the early prediction of the genotypic values of breeding candidates for traits under selection. One of the major limitations of traditional breeding methods is the long generation interval in livestock. In this context, the ability to predict traits targeted for selection in advance contributes significantly to improving the success rate of breeding programmes. This study is significant as it aims to identify the exon regions of the *DDR1* gene associated with performance in Simmental cattle, to define the detected polymorphisms as potential new molecular markers and to facilitate their application in genomic selection programmes for cattle raised in Türkiye. Furthermore, our results may provide valuable data for the scientific literature and journal readers, while also contributing to important advancements in the breeding of dairy cattle with respect to traits influencing both milk yield and quality, which are of great significance for human nutrition.

## 2 Materials and methods

### 2.1 Materials

The material of the study consisted of Simmental breed dairy cattle raised on a private dairy farm in Erzurum province located in the Eastern Anatolia region of Türkiye. Erzurum province is located at 39°55^′^ N latitude and 41°16^′^ E longitude, and the region has a continental climate. In this study, hair samples of 72 Simmental cows of different ages (between 2 and 6 years) and lactation periods (between first and fourth lactations) in a modern free stall barn system with intensive breeding practices were used.

### 2.2 Genomic DNA isolation

Genomic DNA was isolated from the hair samples collected from cattle using the phenol / chloroform + proteinase K method applied by Kiraz (2009) in his study. The extracted DNAs were run on 1 % agarose gel (Bio-Rad), and bands were obtained. Agarose gel electrophoresis was performed by modifying the method of Sambrook et al. (1989). The concentrations and purities of the DNAs visualised were determined to be of sufficient purity by reading on a NanoDrop ND-1000 (NanoDrop Technologies, Inc.) spectrophotometer.

### 2.3 Design of primers

We designed 15 primers for the *DDR1* gene (GenBank accession number NC_037350). The Clone Manager 9 program was used to design the primers. The OligoAnalyzer 3.1 (https://www.idtdna.com/calc/analyzer, last access: 15 February 2019), Clone Manager 9 programs and the equation Tm °C = 2(A + T) + 4(G + C) were used to calculate the Tm values of the primers. The primers designed were purchased from Iontek (Istanbul, Türkiye). Primer sequences of exon regions (5^′^ to 3^′^) are given in Table 1.

**Table 1:** Primer sequences designed for the *DDR1* gene regions.

Gene name	Gene region	Primers (5^′^ to 3^′^)	Size (exon bp)	Tm	GC %	BTA23(NC_037350)
*DDR1*	Exon 1	F: GGCCCTTTCATTTCTACTGCTGCTG	157 bp	64.33	52.00	28184004
		R: GAACACAGGCATATGGAGGGTCAAG		63.39	52.00	28183847
*DDR1*	Exon 3	F: GGTATCCCCTGTGACCTCCT	213 bp	60.2	60.00	28183557
		R: GGAGTAACGCAGTCGGTAGC		59.9	60.00	28183345
*DDR1*	Exon 4	F: CAGGTGATCTTAGGTAATGAGGATC	180 bp	59.73	42.30	28181962
		R: ATGAATTCTTGGGGTCTGAACACTC		61.47	42.00	28181783
*DDR1*	Exon 5	F: GGAGGCTAGAGGTTCTGTGC	155 bp	59.00	60.00	28181595
		R: GCCAACAGTGTGCGTGGTAT		62.00	55.00	28181440
*DDR1*	Exon 6	F: TCTCTTCCAACCTCTTCTTCCTCGG	197 bp	63.54	52.00	28180980
		R: AAACTCAAACTCCATCTCCACGTAG		61.77	46.15	28180783
*DDR1*	Exon 7	F: CACTGCAACAACATGCACAC	243 bp	59.8	50.00	28180539
		R: CGGAAATGAAGGAGATTTCG		59.6	45.00	28180296
*DDR1*	Exon 8	F: CTTTGGTTGCCCCCTTATCT	150 bp	60.30	50.00	28179454
		R: CTCACCCAAGCTGCTGAAGT		60.60	55.00	28179304
*DDR1*	Exon 9	F: ATGGTCCTGACCCTTGTGCT	145 bp	61.9	55.00	28179218
		R: ATGAGGGCAATGATGAGCAG		61.2	50.00	28179073
*DDR1*	Exon 10	F: GTAGGGTATTAGAAGAGGAGCTGAC	226 bp	59.76	48.00	28178228
		R: GAGAGGAAAGGACACACTAGGAAGAC		62.24	50.00	28178002
*DDR1*	Exon 12	F: GGACTATATGGAGCCTGAGAAGCCA	247 bp	63.50	52.00	28176137
		R: GAATCCTACTTACCTCCCCAAACTG		60.63	48.00	28175890
*DDR1*	Exon 13	F: CCTGTCACCTTGGTGAAGCTC	155 bp	62.2	57.10	28175797
		R: GTAGGATCTTGACTGCTACCAACAAA		62.00	42.30	28175642
*DDR1*	Exon 14	F: CAGGGGTGCTAGATTAGAAACTGCAG	190 bp	63.28	50.00	28175516
		R: CGTTCTCCATGTAATCGGTTATCAT		59.07	40.00	28175326
*DDR1*	Exon 15	F: GCTATCTGGCCACACTCAACTTTGT	206 bp	63.50	48.00	28174763
		R: CTCTGTAAGGGCCTCTCACCATAAG		62.19	52.00	28174530
*DDR1*	Exon 16	F: GTCTCTGTCATCTGATGGCTTTCT	153 bp	61.20	45.80	28174054
		R: CTCATCAGTGAGCTGTCCAAAG		60.10	50.00	28173901
*DDR1*	Exon 17	F: CTAAAGACCATTTGCCTGCCTTTCATC	158 bp	62.91	44.44	28173785
		R: CAGGAACCGATGCAACTGGGAAAAG		64.49	52.00	28173627

### 2.4 Polymerase chain reaction (PCR) procedure

The optimum binding temperatures of primers designed specific to *DDR1* gene regions were determined by gradient PCR. PCR reactions were performed in a 200 µ L PCR tube (ViPlus 0.2 mL PCR tube) in a thermal cycler (SensoQuest 48s). For PCR, 1 µ L genomic DNA (50 ng), 4 µ L PCR buffer (10X), 1 µ L of each primer (20 pmol), 1 µ L dNTP (1 mM; D7295; Sigma Aldrich), 0.5 µ L Taq polymerase (D1806; Sigma Aldrich) and 31.5 µ L dH2O to make up to 40 µ L were mixed.

Amplification programmes were as follows: initial denaturation at 95 °C for 5 min, 35 cycles of denaturation at 95 °C for 45 s, 45 s of adhesion (61.2 °C for exons 1^′^ and 3^′^ of the *DDR1* gene; 58.8 °C for exon 4^′^ of the *DDR1* gene; 5^′^, 6^′^, 9^′^, 10^′^, 13^′^ exon regions of the *DDR1* gene at 65.6 °C; for the 12^′^ and 15^′^ exon region of the *DDR1* gene at 67.4 °C; for the 14^′^, 16^′^, 17^′^ exon region of the *DDR1* gene at 63.4 °C), extension at 72 °C for 45 s, followed by a final extension step at 72 °C for 5 min. Finally, the reaction was held at 4 °C.

For gel electrophoresis of PCR products, 1.5 % agarose was dissolved in 1X TBE (90 mM Trizma, 2 mM EDTA, 90 mM boric acid, pH 8.0) buffer solution. 5 µ L of the samples were taken and mixed with 1 µ L of the loading solution (for 10 mL DNA loading buffer; 0.025 mg ( v/v ) bromophenol blue, 40 % sucrose and 2.5 mL EDTA (100 mM) (pH 8.0)). To determine the size of the PCR samples, they were loaded onto the gel with a 100 bp DNA marker (VC 100 bp Plus DNA Ladder; Vivantis) and run under 110 volts for 1 h (Bio-Rad). They were then stained with Et-Br (0.5 µ g mL^−1^; E1510 Sigma Aldrich) for 15 min and visualised under UV.

**Table 2:** Genetic and allele frequencies of polymorphic regions and Hardy–Weinberg equilibrium test results.

Gene region	Marker position	Genotype frequencies (%)			Allele frequencies		H-W	P value
							X2 test	
*DDR1* Exon 1	g.28183947 G/T	GG	GT	TT	G	T	3.46	NS
		0.18 (13)	0.82 (59)	0	0.59	0.41		
*DDR1* Exon 3	g.28183480 G/C	GG	GC	CC	G	C	1.125	NS
		0.78 (56)	0.22 (16)	0	0.76	0.19		
*DDR1* Exon 3	g.28183434 C/A-G	CC	CA	CG	C A G		5.848	NS
		0.60 (43)	0.28 (20)	0.12 (9)	0.76 0.18 0.06			
*DDR1* Exon 5	g.28181525 C/A	CC	CA	AA	C	A	0.97	NS
		0.79 (57)	0.21 (15)	0	0.90	0.10		
*DDR1* Exon 6	g.28180846 G/C	GG	GC	CC	G	C	10.65	NS
		0.44 (32)	0.56 (40)	0	0.72	0.28		
*DDR1* Exon 7	g.28180437 C/G	CC	CG	GG	C	G	6.88	NS
		0.53 (38)	0.47 (34)	0	0.76	0.24		
*DDR1* Exon 10	g.28178065 G/C	GG	GC	CC	G	C	10.65	NS
		0.44 (32)	0.56 (40)	0	0.72	0.28		
*DDR1* Exon 12	g.28175989 G/T	GG	GT	TT	G	T	2.10	NS
		0.71 (51)	0.29 (21)	0	0.85	0.15		
*DDR1* Exon 16	g.28173956 T/G	TT	TG	GG	T	G	5.88	NS
		0.56 (40)	0.44 (32)	0	0.78	0.22		
*DDR1* Exon 16	g.28173954 T/C	TT	TC	CC	T	C	11.41	NS
		0.43 (31)	0.57 (41)	0	0.72	0.28		

NS: non-significant ( P>0.05 ), ^**^ p<0.001 , ^*^ p<0.05 .

### 2.5 Real-time PCR and high-resolution melting (HRM) analysis

HRM analysis was performed on a Rotor-Gene Q real-time PCR device (Qiagen), with different programs for each gene region by applying the protocol in SsoFast EvaGreen^®^ supermix (Bio-Rad). The amplification program consisted of 40 cycles of 98 °C (5 s), 55–65 °C (20 s) and 61–95 °C ( ∞ ) after initial denaturation at 98 °C for the first 2 min. HRM-PCR reactions were performed in 0.1 mL strip tubes (Qiagen). For the reaction, 10 µ L of HRM supermix (1X), 1 µ L each of primers (300–500 nM), 6 µ L of RNase-free water and 2 µ L of template DNA were mixed for a total volume of 20 µ L. Melting curves were obtained as a result of the decrease in fluorescence due to the decrease in fluorescence, with the dye released into the medium as a result of the separation of the double-stranded DNA during the denaturation step of amplification. To determine the differences and similarities between the samples, the samples were divided into clusters using the unsupervised mode according to principal components analysis (Reja et al., 2010) based on the normalisation curves of the samples after amplification with ScreenClust software (Rotor-Gene ScreenClust HRM Software; Qiagen).

### 2.6 DNA sequencing

After the difference between 72 PCR products of each gene region was determined by HRM analysis, the samples were divided into clusters. Four samples were randomly selected from each cluster, and PCR products were sent to a commercial company for sequence analysis.

The references of the PCR products were obtained by using forward/reverse primers of the exon regions amplified from the *Bos taurus* chromosome 23 sequence downloaded from NCBI (https://www.ncbi.nlm.nih.gov, last access: 1 January 2019) to the Clone Manager 9 program. The raw nucleotide sequence chromatograms from the commercial company and the reference sequence of the PCR products were aligned and compared in BioEdit version 7.2.5 (http://www.mbio.ncsu.edu/BioEdit/bioedit.html, last access: 6 January 2020). Genotype differences of the DNA samples were determined by using BioEdit 7.2.5 and ClustalX2.0.4 (http://www.clustal.org/clustal2/, last access: 7 January 2020), ClustalW (http://www.ebi.ac.uk/clustalw, last access: 7 January 2020) and Clone Manager 9 software to convert the sequences into specific formats (FASTA, MEGA) to detect SNPs. Then, amino acid changes of each gene region were determined using MEGA 7 (https://www.megasoftware.net/, last access: 8 January 2020), and polymorphic features were determined using DnaSP version 5 (Rozas, 2009).

### 2.7 Statistical analysis

After the sequences of the DNA samples were aligned and analysed in different programs, SNPs were detected. Then, the detected alleles were distributed to 72 samples according to their clusters. Alleles belonging to each of the cows were added to the Excel file containing the yield records for statistical analysis. After calculating the genotype and allele frequencies of the alleles obtained, the SPSS-26 statistical package program was applied according to the general linear model (Harvey, 1990) to associate polymorphic regions with yield. The data obtained from the study were evaluated using the post hoc Tukey test. The following mathematical model was used in the study:

1 $$Y_{ijkl}=\mu +a_{i}+b_{j}+c_{k}+d_{l}+\varepsilon_{ijklm},$$

where $Y_{ijkl}=$ any yield characteristic (daily average milk yield, 305 d milk yield, lactation milk yield), μ= population mean, $a_{i}=$ impact of genotype, $b_{j}=$ effect of calving season (1: fall, 2: winter, 3: spring, 4: summer), $c_{k}=$ effect of lactation order (k: 2; lactation 1: 1, 2, 3; lactation 2: 4), $d_{l}=$ effect of days open (l: 4, 1: 0–56 d, 2: 57–62 d, 3: 63–90, 4: 91 + d) and $\varepsilon_{ijklm}=$ inclusion as a chance error.

## 3 Results and discussion

### 3.1 PCR results

**Figure 1:**
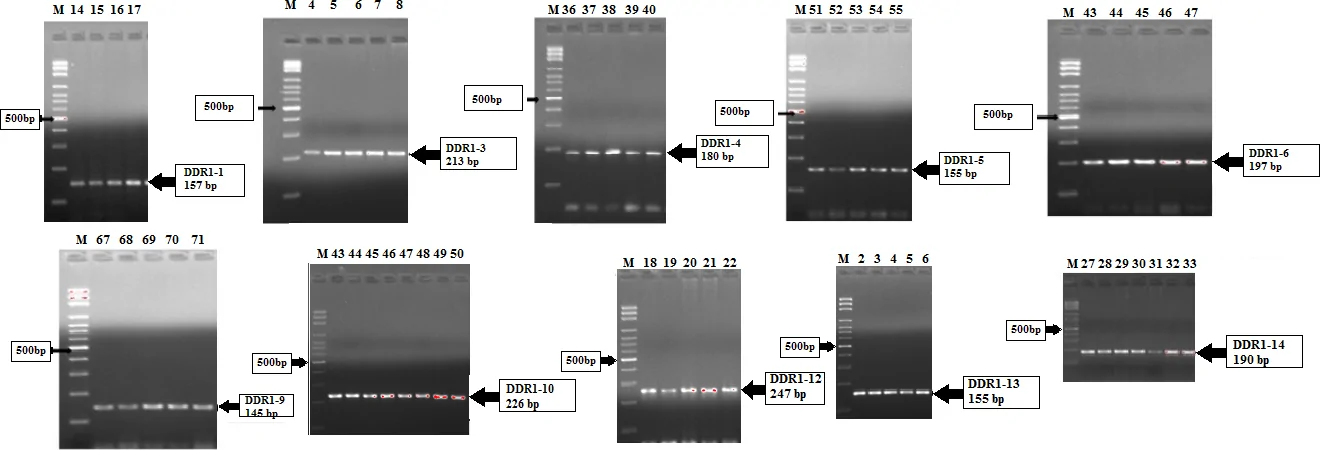
Agarose gel electrophoresis (1.5 %) of PCR products of the DDR1 gene: exon 1 (157 bp), exon 3 (213 bp), exon 4 (180 bp), exon 5 (155 bp), exon 6 (197 bp), exon 9 (145 bp), exon 10 (226 bp), exon 12 (247 bp), exon 13 (155 bp), and exon 14 (190 bp). M: Marker DNA (100 bp).

The PCR amplifications specific to 15 different exon regions of the *DDR1* gene were performed for genomic DNA obtained from Simmental cattle hair samples. PCR products were run on 1.5 % agarose gel, and images were obtained under UV light. The electrophoresis gel images are shown in Fig. 1.

### 3.2 Results of HRM analysis

High-resolution melting (HRM) analysis is a method used in many studies such as mutation screening of sequence variants, genotyping, genetic mapping and candidate gene screening (Nguyen-Dumont et al., 2009). Considering the features of real-time PCR and the advantages of the HRM method, there is growing interest in using the HRM method to screen for sequence variations such as SNPs, micro insertions and deletions (Kuzpınar, 2007). HRM analysis is used to detect diseases associated with mutations and to determine allele prevalence in a population or subgroup (Worm et al., 2001). The HRM method has a procedure that follows the optimised programming of samples in the same tube in a real-time PCR device after the PCR process (Dirican and Akkiprik, 2017). Compared to other imaging methods, HRM analysis is economically recommended because it is simple, fast and cost-effective (Garritano et al., 2009). Due to these advantages, HRM analysis was preferred for use in this study.

**Figure 2:**
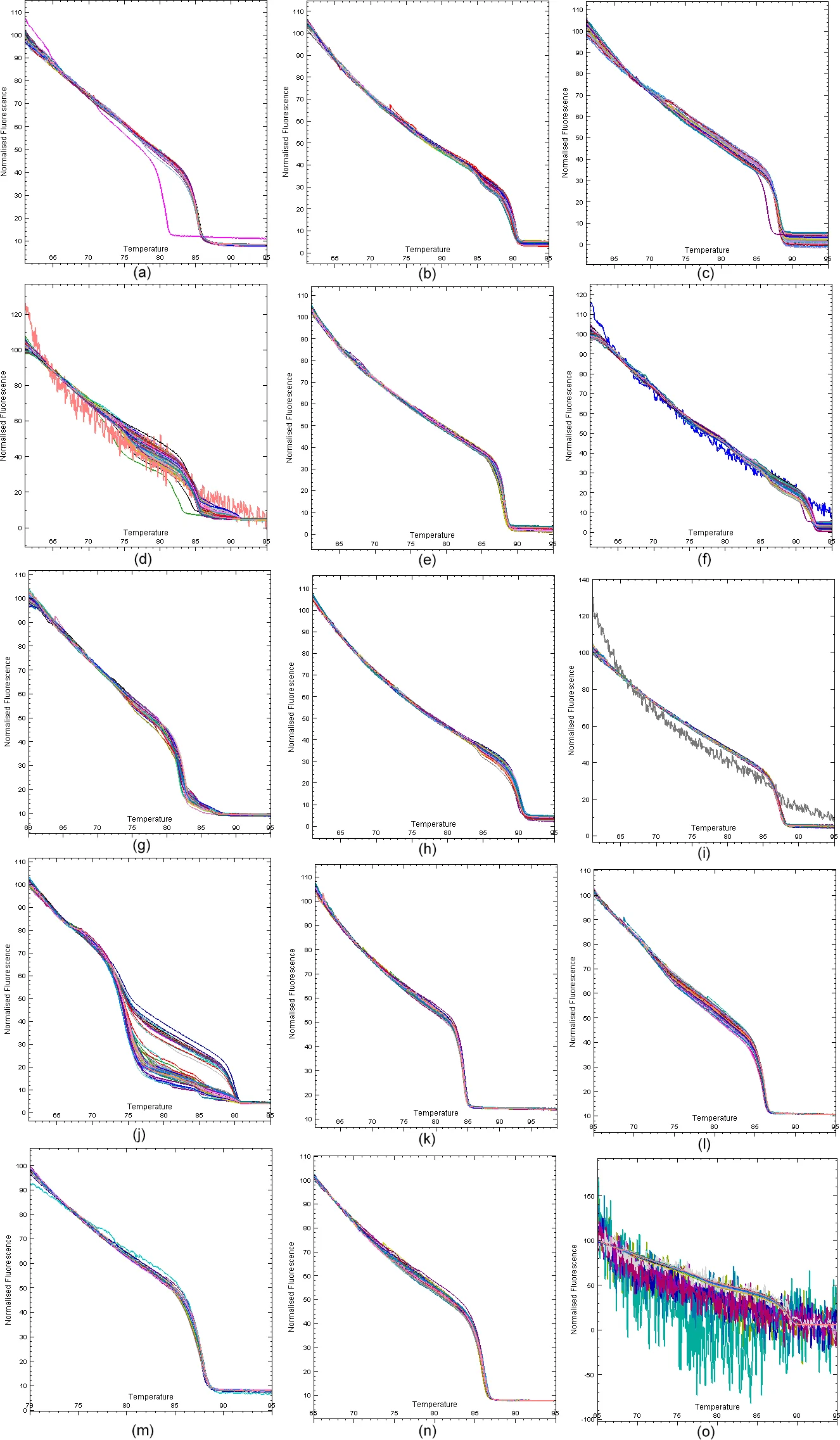
Normalisation HRM curves of *DDR1* gene regions: **(a)** *DDR1* exon 1^′^, **(b)** *DDR1* exon 3^′^, **(c)** *DDR1* exon 4^′^, **(d)** *DDR1* exon 5^′^, **(e)** *DDR1* exon 6^′^, **(f)** *DDR1* exon 7^′^, **(g)** *DDR1* exon 8^′^, **(h)** *DDR1* exon 9^′^, **(i)** *DDR1* exon 10^′^, **(j)** *DDR1* exon 12^′^, **(k)** *DDR1* exon 13^′^, **(l)** *DDR1* exon 14^′^, **(m)** *DDR1* exon 15^′^, **(n)** *DDR1* exon 16^′^ and **(o)** *DDR1* exon 17^′^.

**Figure 3:**
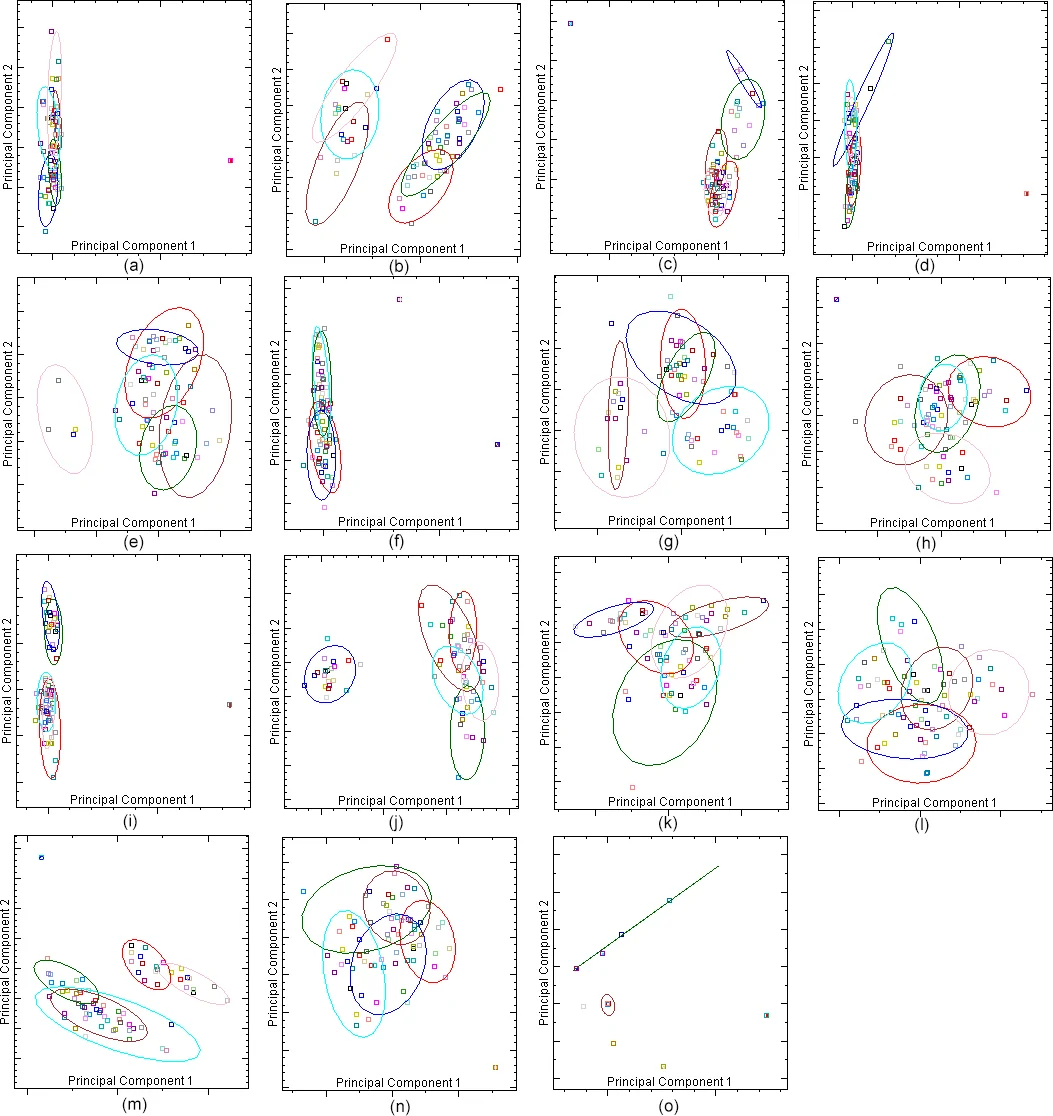
Clusters of principal component analysis of *DDR1* gene regions: **(a)** *DDR1* exon 1^′^, **(b)** *DDR1* exon 3^′^, **(c)** *DDR1* exon 4^′^, **(d)** *DDR1* exon 5^′^, **(e)** *DDR1* exon 6^′^, **(f)** *DDR1* exon 7^′^, **(g)** *DDR1* exon 8^′^, **(h)** *DDR1* exon 9^′^, **(i)** *DDR1* exon 10^′^, **(j)** *DDR1* exon 12^′^, **(k)** *DDR1* exon 13^′^, **(l)** *DDR1* exon 14^′^, **(m)** *DDR1* exon 15^′^, **(n)** *DDR1* exon 16^′^ and **(o)** *DDR1* exon 17^′^.

For each exon region of the DNA samples, HRM analysis was performed at different optimisations using EvaGreen dye, and normalisation curves were obtained (Fig. 2). Samples were clustered according to the melting curve differences of PCR products. By principal component analysis, different numbers of clusters were obtained for each exon region by aggregating dissimilar sequence samples into different clusters. Four randomly selected samples from each cluster were sent to the commercial company for sequencing. The principal component analysis clusters are shown in Fig. 3.

### 3.3 Results of sequence analysis

For each exon region of the *DDR1* gene, the sequence results of the DNA samples and the reference sequence found in NCBI were aligned in the BioEdit 7.2.5 program, and nucleotide variation differences were determined. In addition, genotype and allele frequencies were calculated to determine whether the distribution of genotype frequencies in polymorphic regions was in Hardy–Weinberg equilibrium (Table 3). As a result of the sequence analysis, a total of 10 polymorphic regions were detected in different regions of the *DDR1* gene, including g.28183947 G/T, g.28183480 G/C, g.28183434 C/A-G, g.28181525 C/A, g.28180846 G/C, g.28180437 C/G, g.28178065 G/C, g.28175989 G/T, g.28173956 T/G and g.28173954 T/C.

**Figure 4:**
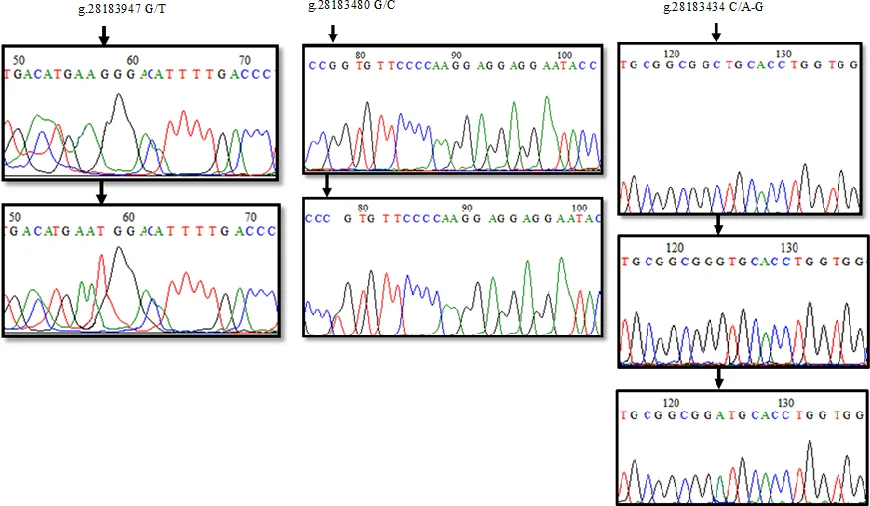
Sequences of polymorphic regions of the 1^′^ and 3^′^ exon of the *DDR1* gene.

The sequence analysis of a total of 21 PCR products obtained from six clusters as a result of HRM analysis in the 1^′^ exon region of the *DDR1* gene was converted into appropriate formats. When the reference sequence of 157 bp nucleotide sequences in the range of 28183847-28184004 of the 1^′^ exon region of the *DDR1* gene was compared with the sequenced samples, six nucleotide differences were detected at position 58 of the sequences. A comparison of the chromatogram peak images of the reference nucleotide sequence of the 1^′^ exon region of the *DDR1* gene and the sequences with nucleotide changes is given in Fig. 4. Accordingly, g.28183947 G/T resulted in GGG → TGG codon change to glycine > tryptophan, and base changes in DNA sequences caused the differentiation of proteins in exon regions. When 24 sequences of 213 bp belonging to the 3^′^ exon region between 28183345 and 28183557 were compared with the reference gene region, four nucleotide differences at position 78 and five nucleotide differences at position 124 were detected (Fig. 4). Accordingly, it was determined that no change occurred in the proline amino acid encoded by CCG → CCC as a result of g.28183480 G/C; on the other hand, for the g.28183434 C/A-G polymorphic region, CTG → ATG codon change caused leucine (L) → methionine (M) amino acid change; CTG → GTG change resulted in leucine (L) → valine (V) amino acid change.

**Figure 5:**
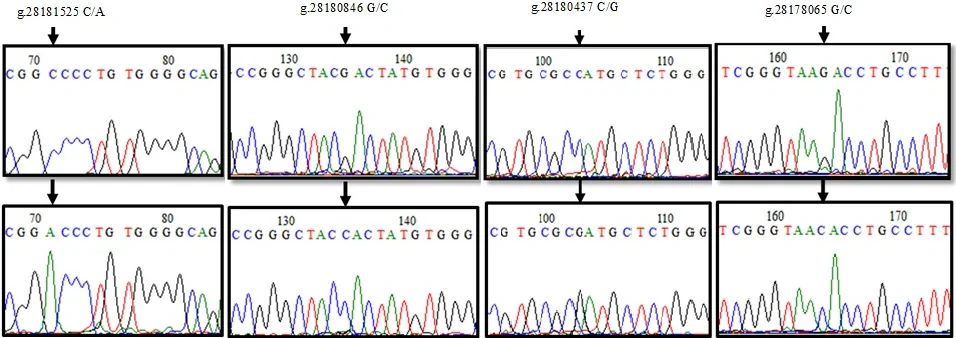
Sequences of the polymorphic regions of exon 5^′^, 6^′^, 7^′^ and 10^′^ of the *DDR1* gene.

When the 155 bp reference sequence NC_037350.1: 28181440–28181595, covering the 5^′^ exon region of *DDR1* gene, was compared with the sequenced PCR products (21), two nucleotide differences were detected at position 71 (Fig. 5). Accordingly, g.28181525 was found to cause alanine-aspartic acid amino acid change as a result of GCC → GAC transversion with C/A. When the reference sequence (197 bp) of the 6^′^ exon region between 28180783 and 28180980 and the sequence results of 24 PCR products were compared, four nucleotide changes were detected at position 135 of the sequences (Fig. 5). Accordingly, the change in the G/C of g.28180846 caused ACG → ACC transversion, but no change occurred in the amino acid threonine. *NC_037350.1*. The sequences of the reference sequence (243 bp) covering the 7^′^ exon region of the *DDR1* gene in the range of 28180296–28180539 and the sequences of the PCR products (18) were aligned in the BioEdit 7.2.5 program, and nucleotide changes were determined (Fig. 5). When the chromatogram peak images were examined, the nucleotide change at position 103 was detected as polymorphic. *g.28180437*. When the C/G polymorphism was examined, it was seen that the histidine amino acid caused the aspartic acid amino acid change as a result of CAT → GAT transversion. When the sequence results of 226 bp PCR samples (21), which were amplified with the sequence in the range of 28178002–28178228 in the 10^′^ exon region, were compared, g.28178065 G/C polymorphism was determined (Fig. 5), and protein change was examined using the MEGA version 7 program. Accordingly, it was determined that arginine (R) was transformed into threonine (T) amino acid as a result of AGA → ACA codon substitution.

**Figure 6:**
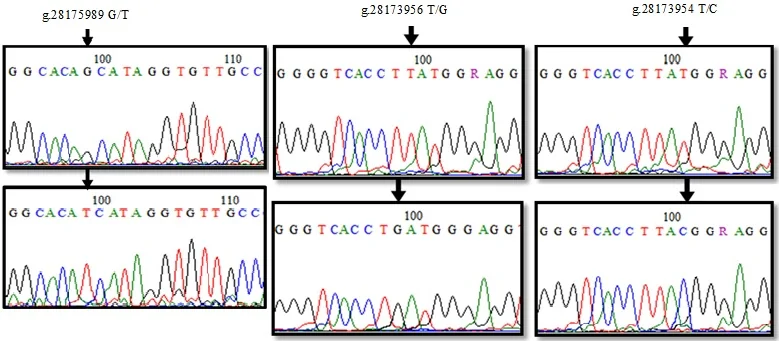
Sequences of the 12^′^ and 16^′^ exon polymorphic regions of the *DDR1* gene.

The nucleotide changes were determined by aligning the sequences of the PCR products (24) amplified with the reference sequence covering the 247 bp region of the 12^′^ exon of the *DDR1* gene in the NC_037350.1: 28175890–28176137 range (Fig. 6). At position 99, the amino acid change of glutamine (Q) → histidine (H) occurred as a result of CAG → CAT transversion in the g.28175989 G/T polymorphic region, while the amino acid glutamine was transformed into histidine amino acid as a result of CAG → CAT transversion, as a result of g.28175989 G/T. When the 153 bp reference sequence of the 16^′^ exon (between 28173901 and 28174054) was compared with the base sequences of 21 PCR products, two different polymorphic regions, g.28173956 T/G and g.28173954 T/C, were identified as a result of base changes at positions 99 and 101 (Fig. 6). g.28173956 with the T/G polymorphism, CTT → CTG transversion occurred, but there was no change in the amino acid leucine (L). g.28173954 with the T/C transversion, ATG → ACG codon change was detected, and, as a result, methionine (M) amino acid was transformed into threonine (T) amino acid.

### 3.4 Genotype and allele frequencies and genetic equilibrium results

The protein variation of the identified polymorphic regions was analysed with the help of the MEGA 7 program. The positions of the polymorphic regions in the genome and their genetic and allelic frequencies are presented in Table 2.

**Table 3:** Mean and standard error values for the polymorphic region at position 58 in the 1^′^ exon of the *DDR1* gene in terms of yield characteristics of Simmental cattle.

	58 (G)	N	Mean	SE mean	P
Daily average milk yield	GG	13	22.72	1.63	NS
	GT	59	20.81	0.73	
Lactation milk yield	GG	13	1993.44	406.95	NS
	GT	59	2224.70	135.03	
305 d milk yield	GG	13	5563.17^a^	317.80	^*^
	GT	59	4934.37^b^	163.98	

^a,b^ Differences between identifiers with different letters in the same column are important. NS: non-significant ( P>0.05 ), ^**^ p<0.001 , ^*^ p<0.05 ; N : number of animals, SE mean: standard error mean.

**Table 4:** Mean and standard error values for the polymorphic region at position 78 in the 3^′^ exon of the *DDR1* gene in terms of yield traits of Simmental cattle.

	78 (G)	N	Mean	SE mean	P
Daily average milk yield	GC	16	20.05	1.22	NS
	GG	56	21.47	0.79	
Lactation milk yield	GC	16	2273.37	251.84	NS
	GG	56	2157.11	154.38	
305 d milk yield	GC	16	4782.98	255.81	NS
	GG	56	5123.60	175.47	

NS: non-significant ( P>0.05 ), ^**^ p<0.001 , ^*^ p<0.05 ; N : number of animals, SE mean: standard error mean.

**Table 5:** Mean and standard error values for the polymorphic region at position 124 in the 3^′^ exon of the *DDR1* gene in terms of yield characteristics of Simmental cattle.

	124 (C)	N	Mean	SE mean	P
Daily average milk yield	CA	20	20.70	1.12	NS
	CC	43	21.19	0.96	
	CG	9	21.99	1.34	
Lactation milk yield	CA	20	2274.80	284.95	NS
	CC	43	2132.18	172.85	
	CG	9	2221.32	226.51	
305 d milk yield	CA	20	4928.31	265.66	NS
	CC	43	5081.88	208.37	
	CG	9	5151.31	296.18	

NS: non-significant ( P>0.05 ), ^**^ p<0.001 , ^*^ p<0.05 ; N : number of animals, SE mean: standard error mean.

### 3.5 Results of statistical analysis

The effects of 72 Simmental cattle genotypes, lactation order, days open and calving seasons on daily average milk yield, lactation milk yield and 305 d milk yield were statistically analysed, and the relationships between these factors and yield traits were determined (Table 13). Upon evaluation of the analysis results, the relationships of the genotypes were indicated separately for each exon region.

#### 3.5.1 Association analysis of *DDR1* gene 1^′^ exon region with milk yield

The relationships between the g.28183947 G/T polymorphic region determined in the 157 bp 1^′^ exon region and daily average milk yield, lactation milk yield and 305 d milk yield were analysed by analysis of variance. According to the results of the analysis, there was no significant relationship between the g.28183947 G/T polymorphic region and daily average milk yield and lactation milk yield, but there was a statistically significant relationship between the 305 d milk yield values ( P<0.05 ). Indeed, in a study conducted to investigate the effect of the *DDR1* gene on apoptosis, proliferation and cell cycle of mammary epithelial cells in dairy cows, pcDNA3.1-*DDR1*, a small RNA blocker fragment of the *DDR1* gene and overexpression vector was transfected into mammary epithelial cells of dairy cows. The results of the study showed that when the *DDR1* gene was interfered with in dairy cow mammary epithelial cells, the mRNA and protein expression levels of *DDR1* were down-regulated by 94 % and 30 %, respectively. After overexpression of the *DDR1* gene, mRNA and protein expression were found to be up-regulated 68.24- and 1.38-fold, respectively. Therefore, it was understood that *DDR1* can regulate proliferation, apoptosis and cycling of dairy cow mammary epithelial cells (Hua et al., 2020). In another study supporting our findings, it was stated that *DDR1* is a central gene of the miRNA regulatory network responsible for controlling milk yield in cows (Do et al., 2017). The means and standard errors for the yield traits associated with the *DDR1* gene 1^′^ exon region are given in Table 3.

**Table 6:** Mean and standard error values for the polymorphic region at position 71 in the 5^′^ exon of the *DDR1* gene in terms of yield traits of Simmental cattle.

	71 (C)	N	Mean	SE mean	P
Daily average milk yield	CA	15	23.73^a^	1.53	^*^
	CC	57	20.48^b^	0.72	
Lactation milk yield	CA	15	2198.15^a^	331.33	^*^
	CC	57	2178.94^b^	143.51	
305 d milk yield	CA	15	5692.28^a^	328.17	^*^
	CC	57	4878.33^b^	159.79	

^a,b^ Differences between identifiers with different letters in the same column are important. NS: non-significant ( P>0.05 ), ^**^ p<0.001 , ^*^ p<0.05 ; N : number of animals, SE mean: standard error mean.

**Table 7:** Mean and standard error values for the polymorphic region at position 135 in the 6^′^ exon of the *DDR1* gene in terms of yield traits of Simmental cattle.

	135 (G)	N	Mean	SE mean	P
Daily average milk yield	GC	40	21.56	0.93	NS
	GG	32	20.66	0.96	
Lactation milk yield	GC	40	2130.04	164.24	NS
	GG	32	2249.07	216.43	
305 d milk yield	GC	40	5079.52	211.25	NS
	GG	32	5008.38	206.44	

NS: non-significant ( P>0.05 ), ^**^ p<0.001 , ^*^ p<0.05 ; N : number of animals, SE mean: standard error mean.

#### 3.5.2 Association analysis of *DDR1* gene 3^′^ exon region with milk yield

The relationships between g.28183480 G/C and g.28183434 C/A-G polymorphic regions detected in the 3^′^ exon region of the *DDR1* gene and daily average milk yield, lactation milk yield and 305 d milk yield were analysed statistically. As a result of the analyses, it was found that the effect of the genotypes of the g.28183480 G/C polymorphism at position 78 of the 3^′^ exon region of the *DDR1* gene on daily average milk yield was not significant ( P>0.05 ), the effect on lactation milk yield was insignificant ( P>0.05 ) and the effect on 305 d milk yield was also insignificant ( P>0.05 ). The effect of the genotypes of g.28183434 C/A-G polymorphism at position 124 of the 3^′^ exon region on daily average milk yield was found to be insignificant ( P>0.05 ), the effect on lactation milk yield was found to be insignificant ( P>0.05 ) and the effect on 305 d milk yield was also found to be insignificant ( P>0.05 ). The results of the means and standard errors of the yield traits of the *DDR1* gene 3^′^ exon region are given in Tables 4 and 5.

#### 3.5.3 Association analysis of *DDR1* gene 5^′^ exon region with milk yield

The relationships between the g.28181525 C/A polymorphic region detected in the 5^′^ exon region of the *DDR1* gene and daily average milk yield, lactation milk yield and 305 d milk yield were statistically calculated. According to the results of the analysis, it was found that the effect of g.28181525 C/A polymorphism at position 71 of the 5^′^ exon region of the *DDR1* gene (155 bp) on daily average milk yield was significant ( P<0.05 ), the effect on lactation milk yield was significant ( P<0.05 ) and the effect on 305 d milk yield was also significant ( P<0.05 ).

In a study on milk formation of the *DDR1* gene in animals, the importance of *DDR1* was mentioned by inactivating the gene encoding *DDR1* in mice. *DDR1* binding sites were identified by staining mouse sections with the extracellular domain of *DDR1* conjugated with alkaline phosphatase. To analyse the role of *DDR1* in vivo, a deletion was introduced into the *DDR1* gene in the mouse germ line. After the intervention, mice lacking *DDR1* were small, and mutant females showed multiple reproductive defects. Consequently, most mutant females failed to give birth as the developing blastocysts did not implant. Mutant females that gave birth were unable to feed their offspring because the mammary gland epithelium failed to secrete milk. Histological analysis showed that the alveolar epithelium was unable to secrete milk proteins into the lumen of the mammary gland. Thus, it was inferred that the lactation disorder was caused by hyperproliferation and abnormal branching of the mammary ducts. These results suggest that *DDR1* is an important mediator of stromal–epithelial interaction during ductal morphogenesis in the mammary gland. The authors stated that *DDR1* is an important mediator of stromal–epithelial interaction during ductal morphogenesis in the mammary gland (Vogel et al., 2001).

In another study investigating the indirect effect of *DDR1* on milk yield, it was reported by weighted gene co-expression network analysis (WGCNA) that *DDR1* plays a role in mammary duct morphogenesis in milk fat metabolism in dairy cattle and thus has an important effect on milk fat synthesis (Mu et al., 2022). Again, as a result of mammary tissue transplantation studies on mice, it was reported that *DDR1* signalling is the third matrix-derived pathway in addition to hormone and cytokine receptors in the maintenance of mammary gland functions (Faraci-Orf et al., 2006). The results of the means and standard errors of the yield traits of the *DDR1* gene 5^′^ exon region are given in Table 6.

**Table 8:** Mean and standard error values for the polymorphic region at position 103 in the 7^′^ exon of the *DDR1* gene in terms of yield traits of Simmental cattle.

	103 (C)	N	Mean	SE mean	P
Daily average milk yield	CC	38	21.01	0.96	NS
	CG	34	21.33	0.94	
Lactation milk yield	CC	38	2359.11	180.93	NS
	CG	34	1986.05	189.47	
305 d milk yield	CC	38	5084.30	195.28	NS
	CG	34	5007.22	227.86	

NS: non-significant ( P>0.05 ), ^**^ p<0.001 , ^*^ p<0.05 ; N : number of animals, SE mean: standard error mean.

**Table 9:** Mean and standard error values for the polymorphic region at position 164 in the 10^′^ exon of the *DDR1* gene in terms of yield traits of Simmental cattle.

	164 (G)	N	Mean	SE mean	P
Daily average milk yield	GC	40	20.79	0.93	NS
	GG	32	21.61	0.97	
Lactation milk yield	GC	40	2167.85	186.07	NS
	GG	32	2201.81	187.22	
305 d milk yield	GC	40	4967.17	212.25	NS
	GG	32	5148.83	203.59	

NS: non-significant ( P>0.05 ), ^**^ p<0.001 , ^*^ p<0.05 ; N : number of animals, SE mean: standard error mean.

#### 3.5.4 Association analysis of *DDR1* gene 6^′^ exon region with milk yield

The relationships between the g.28180846G/C polymorphism in the 6^′^ exon region and daily average milk yield, lactation milk yield and 305 d milk yield were statistically calculated. When statistical analyses were evaluated, it was found that the effect of g.28180846G/C polymorphism at position 135 of the 6^′^ exon of the *DDR1* gene on daily average milk yield was insignificant ( P>0.05 ), the effect on lactation milk yield was insignificant ( P>0.05 ) and the effect on 305 d milk yield was also insignificant ( P>0.05 ). The results of the means and standard errors of the means for the yield traits of the *DDR1* gene 5^′^ exon region are given in Table 7.

#### 3.5.5 Association analysis of *DDR1* gene 7^′^ exon region with milk yield

The relationships between the g.28180437 C/G polymorphic region defined in the 7^′^ exon region of the *DDR1* gene and daily average milk yield, lactation milk yield and 305 d milk yield were analysed by analysis of variance. Accordingly, the effect of g.28180437 C/G polymorphism at position 103 on daily average milk yield was insignificant ( P>0.05 ), on lactation milk yield was insignificant ( P>0.05 ) and on 305 d milk yield was also insignificant ( P>0.05 ). The results of the means and standard errors of the means for the yield traits of the *DDR1* gene 7^′^ exon region are given in Table 8.

#### 3.5.6 Association analysis of *DDR1* gene 10^′^ exon region with milk yield

When the relationships between the g.28178065 G/C polymorphic region in the 10^′^ exon region and daily average milk yield, lactation milk yield and 305 d milk yield were analysed statistically, the effect of g.28178065 at position 164 and the effect of G/C polymorphism on daily average milk yield of all Simmental cattle was found to be insignificant ( P>0.05 ), the effect on lactation milk yield was found to be insignificant ( P>0.05 ) and the effect on 305 d milk yield was also found to be insignificant ( P>0.05 ). The mean and standard error values for yield traits are given in Table 9.

#### 3.5.7 Association analysis of *DDR1* gene 12^′^ exon region with milk yield

The relationships between the g.28175989 G/T polymorphism in the 247 bp long amplified region covering the 12^′^ exon of the *DDR1* gene and daily average milk yield, lactation milk yield and 305 d milk yield were analysed. Accordingly, the effect of g.28175989 G/T polymorphism at position 99 on daily average milk yield was found to be insignificant ( P>0.05 ), the effect on lactation milk yield insignificant ( P>0.05 ) and the effect on 305 d milk yield also insignificant ( P>0.05 ). The results of the means and standard errors for the yield traits of the *DDR1* gene 12^′^ exon region are given in Table 10.

**Table 10:** Mean and standard error values for the polymorphic region at position 99 in the 12^′^ exon of the *DDR1* gene in terms of yield traits of Simmental cattle.

	99 (G)	N	Mean	SE mean	P
Daily average milk yield	GG	51	20.58	0.84	NS
	GT	21	22.57	1.01	
Lactation milk yield	GG	51	2088.81	170.19	NS
	GT	21	2411.55	178.56	
305 d milk yield	GG	51	4904.49	184.64	NS
	GT	21	5396.21	225.97	

NS: non-significant ( P>0.05 ), ^**^ p<0.001 , ^*^ p<0.05 ; N : number of animals, SE mean: standard error mean.

#### 3.5.8 Association analysis of *DDR1* gene 16^′^ exon region with milk yield

The relationships between g.28173956 T/G and g.28173954 T/C polymorphic regions and daily average milk yield, lactation milk yield and 305 d milk yield were analysed statistically. Genotypes of g.28173956 T/G polymorphism at position 99 had an insignificant effect on daily average milk yield ( P>0.05 ), an insignificant effect on lactation milk yield ( P>0.05 ) and an insignificant effect on 305 d milk yield ( P>0.05 ). Genotypes belonging to g.28173954 T/C polymorphic region at position 101 had an insignificant effect on daily average milk yield ( P>0.05 ), an insignificant effect on lactation milk yield ( P>0.05 ) and an insignificant effect on 305 d milk yield ( P>0.05 ). The results of the means and standard errors of the means for the yield traits of the *DDR1* gene 16^′^ exon regions are given in Tables 11 and 12.

## 4 Conclusions

As a result, 10 polymorphic regions were detected for the first time in the *DDR1* gene, including g.28183947 G/T in the 1^′^ exon region of the *DDR1* gene, g.28183480 G/C and g.28183434 C/A-G in the 3^′^ exon region, g.28181525 C/A in the 5^′^ exon region, g.28180846 G/C in the 6^′^ exon region, g.28180437 in the 7^′^ exon region C/G, g.28178065 G/C in the 10^′^ exon region, g.28175989 G/T in the 12^′^ exon region, and g.28173956 T/G and g.28173954 T/C in the 16^′^ exon region. The effects of these polymorphic regions on daily average milk yield, lactation milk yield and 305 d milk yield were statistically calculated. As a result of the statistical analysis, it was found that g.28183947 G/T polymorphism in the 1^′^ exon region had a significant effect on 305 d milk yield ( P<0.05 ). Meanwhile, g.28181525 C/A polymorphism in the 5^′^ exon region had a significant effect on 305 d milk yield ( P<0.05 ), a significant effect on daily average milk yield ( P<0.05 ) and a significant effect on lactation milk yield ( P<0.05 ).

**Table 11:** Mean and standard error values for the polymorphic region at position 99 in the 16^′^ exon region of the *DDR1* gene in terms of yield characteristics of Simmental cattle.

	99 (T)	N	Mean	SE mean	P
Daily average milk yield	TG	32	22.10	0.93	NS
	TT	40	20.41	0.94	
Lactation milk yield	TG	32	2104.41	187.19	NS
	TT	40	2245.77	185.51	
305 d milk yield	TG	32	5166.85	227.71	NS
	TT	40	4952.75	195.68	

NS: non-significant ( P>0.05 ), ^**^ p<0.001 , ^*^ p<0.05 ; N : number of animals, SE mean: standard error mean.

**Table 12:** Mean and standard error values for the polymorphic region at position 101 in the 16^′^ exon region of the *DDR1* gene in terms of yield characteristics of Simmental cattle.

	101 (T)	N	Mean	SE mean	P
Daily average milk yield	TC	41	20.88	1.00	NS
	TT	31	21.53	0.82	
Lactation milk yield	TC	41	2001.34	179.54	NS
	TT	31	2423.12	187.79	
305 d milk yield	TC	41	4936.01	214.30	NS
	TT	31	5195.90	195.31	

NS: non-significant ( P>0.05 ), ^**^ p<0.001 , ^*^ p<0.05 ; N : number of animals, SE mean: standard error mean.

**Table 13:** Mean and standard error values of the effects of Simmental cattle genotype, lactation order, number of days open to vaccination and calving seasons on average daily milk yield, lactation milk yield and 305 d milk yield.

		Group	N	Mean	SE mean	P
Daily average milk yield	Calving season	1	23	20.42^ab^	0.93	^*^
		2	39	23.11^a^	0.90	
		3	4	16.18^b^	3.07	
		4	6	14.64^b^	0.90	
	Lactation order	1	36	19.03^b^	0.95	^*^
		2	36	23.29^a^	0.81	
	Days open	1	18	22.56	1.62	NS
		2	20	21.25	1.33	
		3	17	21.87	1.07	
		4	17	18.86	1.14	
Lactation milk yield	Calving season	1	23	2921.65^a^	142.99	^*^
		2	39	1559.21^b^	113.27	
		3	4	2419.69^ab^	1410.62	
		4	6	3247.65^a^	164.53	
	Lactation order	1	36	2054.59^b^	209.70	*
		2	36	2311.29^a^	159.88	
	Days open	1	18	1184.48^c^	231.82	^**^
		2	20	2100.54^b^	197.57	
		3	17	2275.92^b^	165.42	
		4	17	3244.11^a^	216.16	
305 d milk yield	Calving season	1	23	5071.52^ab^	223.63	^*^
		2	39	5335.28^a^	209.58	
		3	4	3676.07^b^	476.76	
		4	6	4003.99^ab^	227.59	
	Lactation order	1	36	4523.16^b^	208.96	^*^
		2	36	5572.65^a^	171.68	
	Days open	1	18	5157.53	388.06	NS
		2	20	5006.45	289.67	
		3	17	5184.73	249.60	
		4	17	4843.77	240.96	

^a,b,c^ Differences between identifiers with different letters in the same column are important. NS: non-significant ( P>0.05 ), ^**^ p<0.001 , ^*^ p<0.05 ; N : number of animals, SE mean: standard error mean.

With this study, it is suggested that the polymorphic regions detected on the *DDR1* gene of cattle can be used as genetic markers in cattle breeding, and, since it is a study in which the relevant gene regions are examined at the molecular level together with HRM analysis, it may serve as a reference for scientists who will study the subject. It is recommended that similar studies should be carried out in various dairy cattle breeds raised in different regions in different *DDR1* gene regions that have been found to have an effect on milk yield. Such molecular genetic studies may provide important gains regarding breeding some traits for milk yield and quality in dairy cattle breeding.

Since there were no previous studies on the *DDR1* gene in cattle including SNP and sequence analysis, the comparison of the obtained polymorphic regions with the literature supported the expression level studies on *DDR1* gene milk yield.

## Data Availability

The datasets are available upon request from the corresponding author.
